# Who are Adults with Developmental Language Disorder? Profiles from the Engage with Developmental Language Disorder Project

**DOI:** 10.1177/23969415261461413

**Published:** 2026-08-10

**Authors:** Jenny L Gibson, Nicola Botting, Ellie Barker, Emily M Jackson, Suze Leitão, Michelle C St Clair

**Affiliations:** 1 Faculty of Education, 2152 University of Cambridge, Cambridge, UK; 2 School of Health and Medical Sciences, City St George's, University of London, London, UK; 3 Department of Psychology, 1555 University of Bath, Bath, UK; 4 Curtin School of Population Health, enAble Institute, 1649 Curtin University, Perth, Australia; 5 Curtin School of Allied Health, enAble Institute, 1649 Curtin University, Perth, Australia

**Keywords:** Developmental language disorder, adulthood, profile, mental health

## Abstract

**Background and Aims:**

Although developmental language disorder (DLD) is a lifelong, neurodevelopmental condition, its presentation in adulthood has received scant research and clinical attention. Several longitudinal studies have demonstrated that poor language ability may be negatively associated with wider life factors in adulthood. Moreover, although a growing body of qualitative evidence illustrates the lived experience of adults with DLD, more quantitative research on this subject is needed, which may guide evidence-informed assessment and support practices for adults. In this short report, we aim to explore the presentation of DLD in adulthood. We share the demographic characteristics, language difficulties, and mental health profile of a sample of adults with DLD based on self-report data.

**Methods:**

We collected data from 92 adult participants recruited via Engage with Developmental Language Disorder (E-DLD), an international database set up to facilitate DLD research. Participants completed a DLD screening questionnaire, a set of demographic questions, and measures of emotional health including anxiety, depression, and self-esteem. We used the resulting data to construct an overview of the characteristics and profiles of two subgroups in the database: (a) adults who self-reported a clinical diagnosis of DLD (n = 30); and (b) adults who scored highly on the screening questionnaire (“possible DLD,” n = 62) but who did not have a formal diagnosis. For some measures, we also include a comparative sample of typical adults with no confirmed or suspected DLD (n = 91).

**Results:**

More women than men completed the survey, with the average age of the DLD participants being just under 37 years of age, whereas the controls were on average 43 years of age. About 70% of the DLD participants were UK residents, while 30% were from countries across the world. Participants with DLD tended to be lower earners than controls but were represented in all income bands. In total, 43% (39) were parents and 43% (39) were living with a partner. Word finding and expressive language were the most reported areas of language difficulty, and the clinically diagnosed sample reported significantly more pragmatic language challenges than the “possible DLD” group. Respondents across both DLD groups had higher levels of emotional challenges including anxiety, depression, alexithymia, and low self-efficacy compared to the comparison sample.

**Conclusions:**

Adults with DLD are a diverse group, with potentially more women than men seeking diagnosis or recognition in adulthood. It is possible that the high rates of emotional difficulties may indicate notable levels of need in those with DLD in adulthood.

**Implications:**

Our findings show that there are many adults with likely DLD without a formal diagnosis. As such, they are unlikely to qualify for support. We recommend that services providing for adults with DLD should make every effort to be accessible to those on lower incomes, given our income findings, and services could usefully collaborate with mental health services to understand the complexities of co-occurring needs. Speech and language services should also consider how to work alongside other advocates to increase recognition of DLD in adulthood, given the lifelong impact of DLD, whether diagnosed or not.

## Introduction

Developmental language disorder (DLD) is characterized by developmental onset of enduring difficulties in acquisition, comprehension, production or use of language, that are not better explained by other factors, are unlikely to resolve spontaneously and that impact on daily life (Bishop et al., 2017). The language profile of individuals with DLD is variable and multidimensional, including the issues discussed above as well as pragmatic and semantic difficulties (McGregor et al., 2025). In the major diagnostic classification systems (DSM-5, ICD-11.00), it is classed as a neurodevelopmental disorder, meaning it has its onset in the childhood period and is linked to fundamental differences in neurological development and everyday functioning (American Psychiatric Association, 2013; WHO, 2019). Like all neurodevelopmental disorders, therefore, DLD is not a transient phenomenon but a lifelong condition (Whitehouse et al., 2009). However, to date, both academic research and clinical service provision for those with DLD are heavily skewed toward children (Wilmot et al., 2024). This means that there are many adults with DLD who do not receive understanding, accommodations, and/or support for their condition. Furthermore, with childhood diagnosis rates that are significantly lower than population prevalence estimates, many adults affected by DLD will not have had a diagnosis or access to support at any time in their lives (McGregor, 2020).

The consequences of a lack of recognition and support are potentially serious. Longitudinal studies based on samples drawn from population cohorts have demonstrated that low language ability is a risk factor for poor mental health, academic attainment, employment, and quality of life outcomes (e.g., Eadie et al., 2018; Nudel et al., 2023; Ziegenfusz et al., 2022). Similar findings are reported in those studies following children diagnosed with DLD^1^ in childhood (see Dubois et al., 2020 for a review). For example, in the UK, The Manchester Language Study (https://reshare.ukdataservice.ac.uk/852066/) followed a group of 242 children with DLD (recruited at 7), and a typically developing control group (recruited at 16) through adolescence and into adulthood, measuring language and wider lifestyle impacts. At age 24 years, the DLD group had higher levels of emotional need and lower levels of vocational and academic attainment (Botting et al., 2016; Conti-Ramsden et al., 2018). Although employment rates were comparable to those of the controls, those with language difficulties were more likely to be in part-time work in non-professional roles.

In a study with a similar design, based in Canada, 31-year-old adults with a history of DLD were found to be more likely to have lower incomes than adults with histories of typical language (Beitchman et al., 2014). Interestingly, although rates of psychiatric disorder at age 31 were similar to those of controls, the researchers found a higher risk of psychiatric disorder when the participants were in late adolescence/early adulthood (Beitchman et al., 2001). This mismatch may be due to non-random attrition in their study, or could reflect that those with DLD become more adept at finding necessary support as they age, or that difficulties decrease after the early adulthood stage.

Qualitative research provides us with insight into how these findings translate into the lived experience of adults with DLD. Wilmot et al. (2024) found that adults with DLD experienced isolation and loneliness as well as frustration and a lack of understanding in the workplace. Echoing the findings from quantitative research as described above (e.g., Botting et al., 2016; Conti-Ramsden et al., 2018), Wilmot et al. (2024) also identified a lived experience of academic difficulties among adults with DLD for a range of academic and socioemotional reasons, with transitions from secondary to post-secondary school environments highlighted as being particularly challenging. Importantly, this study also found that adults with DLD see enormous value in the peer and community support afforded by connecting with those with similar struggles.

Together, both qualitative and quantitative evidence show that recognition and support for those with DLD should extend into adulthood. As Hobson and colleagues (Hobson et al., 2024) have observed, research and practice in DLD have yet to catch up to the levels of attention given to autism and ADHD, where neurodiversity-based approaches have supported adults seeking late diagnoses and sparked research efforts into lifespan experiences. We believe that an important part of moving forward from the current situation will be for the emerging research community around adult DLD to be proactive and open, sharing information and analyses as they become available.

### The Present Report

This short report aims to contribute to this emerging field by sharing insights into the presentation of adult DLD. We share descriptive summaries of the demographic characteristics, language difficulties, and mental health profile of a sample of adults with DLD based on self-report data. We also provide some statistical comparisons on these measures to a sample of adults reporting typical language development (TLD); we considered these comparisons to be exploratory and did not test specific directional hypotheses. We interpret the findings in the light of our clinical and academic experience as well as with reference to relevant research literature. We hope that by sharing initial insights and data, this paper will contribute to the small but emerging research base, support clinicians working with adults with DLD, and inform the DLD community about the strengths and challenges that may be experienced in adulthood.

## Method

This project received ethical approval through the University of Bath Department of Psychology Research Ethics committee (REF: 20-207 and 20-208). All participants gave informed written consent after reading an online information sheet about the study.

### Approach

To meet our aim of sharing useful profiling information for research and clinical use, we analyzed data from the Engage with Developmental Language Disorder project (E-DLD; St Clair et al., 2023; www.engage-dld.com). The E-DLD project is an international database supporting research into DLD in children and adults (see St Clair et al. (2023) for details).

The E-DLD database is regularly advertised for sign-ups via social media and relevant online communities. All E-DLD members voluntarily sign-up for the project. At sign-up, 32.6% were under 30, 51.1% were between 30 and 50, and 16.3% were over 50. Clinicians are encouraged to share E-DLD, and there are regular seminars communicating the purpose and nature of the E-DLD project, which is guided by a yearly member consultation. While the database was initiated in the UK, E-DLD's partnerships with DLD support organizations in the US and Australia have contributed to the database attracting increasing numbers of international sign-ups. All E-DLD members provide informed consent for their data to be anonymized and used for research purposes.

### Recruitment and Eligibility

For the present study, we included adults with a confirmed clinical diagnosis of DLD (“Diagnosed DLD”) who were enrolled in the E-DLD database. We also recruited adults without a confirmed clinical diagnosis but who suspected they had DLD (“Possible DLD”). The latter group completed a screening questionnaire (see below) that is currently being developed and validated by the present authors (St Clair et al., in preparation). Only individuals who self-reported pervasive long-term language needs, either through diagnosis or within our screening questionnaire, were included in the present dataset.

We used the Prolific data collection service to recruit 100 TLD adults to complete selected study measures and serve as a comparison sample. The Prolific data collection service is an online database of participants, who can choose to take part in studies advertised on this website in exchange for payment. We recruited only UK residents, as we found in previous studies that global recruitment leads to very skewed samples with 40–50% of the sample from a single low and middle income country (Stoyanova et al., under review). Recruiting globally would not have replicated the broad distribution of countries in the E-DLD database; therefore, we restricted this comparison sample to the UK.

The data comprised information from 92 adults with DLD who had signed up to the E-DLD database. Thirty of these adults had “Diagnosed DLD” and 62 had “Possible DLD” as indicated by our screening tool. Our TLD comparison sample was reduced from 100 to 91, as we excluded eight participants whose scores on the screening tool overlapped with the range in the E-DLD sample, and one participant who reported a diagnosis of aphasia.

Table 1 provides an overview of the demographic characteristics of our participants. Please see Supplementary Materials for an equivalent table looking at only the reduced sample with yearly surveys. Most of the sample was based in the UK, with more females than males completing the survey. Most participants identified as “White” (75% of the total DLD group; 83.5% of the TLD group), with a smaller group reporting they identified as “Asian” (5.4% for DLD; 7.7% for TLD). Around 10% of the DLD group preferred not to share this information.

**Table 1.** Demographic Information for Those with Diagnosed DLD, Possible DLD, the Combined DLD Group and TLD Comparison Group.

	Diagnosed DLD (N = 30)	Possible DLD (N = 62)	Total DLD (N = 92)	TLD Comparison (N = 91)
Gender^a^ (N (% female))	26 (86.7%)	47 (75.8%)	73 (79.4%)	60 (65.9%)
Age (M, SD)	36 (13.00)	37.19 (12.22)	36.80 (12.42)	42.83 (13.6)
Ethnicity (N, %)				
White	27 (90.0%)	42 (67.7%)	69 (75.0%)	76 (83.5%)
Asian	–	5 (8.1%)	5 (5.4%)	7 (7.7%)
Black	–	3 (4.8%)	3 (3.3%)	3 (3.3%)
Mixed	2 (6.7%)	2 (3.2%)	4 (4.4%)	3 (3.3%)
Hispanic	–	3 (4.8%)	3 (3.3%)	1 (1.1%)
Other, not reported	1 (3.3%)	7 (11.3%)	8 (8.7%)	1 (1.1%)
Bilingual status (N, %)				
Monolingual	27 (90.0%)	47 (75.8%)	74 (80.4%)	80 (87.9%)
Bilingual	2 (6.7%)	11 (17.7%)	13 (14.1%)	11 (12.1%)
Multilingual	1 (3.3%)	4 (6.5%)	5 (5.4%)	–
UK resident^b^ (N, %)	23 (76.7%)	40 (66.7%)	63 (70.0%)	91 (100%)

*Note.* DLD = developmental language disorder; TLD = typical language development.

a The remaining sample were male, except for 1 (1.4%) “prefer not to say” within the Possible DLD group

b Two participants in the possible DLD group have missing data on the UK residency variable.

**Table 2.** Family Circumstances of Adults with DLD.

	Diagnosed DLD (N = 30)	Possible DLD (N = 61)	Total DLD (N = 91)
Parent (N,%)^a^	13 (43.3%)	26 (43.3%)	39 (43.3%)
Number of children (M, SD)	1.92 (.86)	2.04 (1.00)	2 (.95)
**Relationship status**			
Living with partner	7 (23.3%)	32 (52.5%)	39 (42.9%)
Separated/Single	17 (56.7%)	25 (41.0%)	42 (46.2%)
Dating/ living apart	5 (16.7%)	4 (6.6%)	9 (9.9%)
Other	1 (3.3%)	–	1 (1.1%)

*Note.* DLD = developmental language disorder.

a Sample size for this variable was 90 in total, with 1 possible DLD participant opting not to respond.

### Measures

All adults with DLD (“Diagnosed DLD” and “Possible DLD”) completed the sign-up questionnaire and were invited to complete the yearly survey. Participants with TLD completed only the yearly survey, with the addition of the Screening Tool for Adults with Non-Diagnosed DLD (STAND, which was also administered to the Possible DLD group) and select demographic questions from the sign-up survey.

#### Screening Tool for Adults with Non-Diagnosed Developmental Language Disorder

As no validated screening tool is yet available for adults with DLD, the team created a screening tool based on their expert knowledge. Broadly the questionnaire asks about history of language difficulties, the nature of any language difficulties, the impact of any difficulties on everyday life, other diagnoses, family history of relevant conditions and questions to rule out confounding reasons for any language difficulties (e.g., stroke, head injury). We have not reported exact items as the screening tool development process is ongoing and subject to embargo (St Clair et al., in preparation).

#### Sign-up Questionnaire

Alongside the STAND screening tool administered to only those without a formal DLD diagnosis (e.g., the possible DLD group), our DLD participants also completed a standard E-DLD database of sign-up questions in the following domains:

**Demographics.** Participants reported on gender, ethnicity, languages spoken, age, relationship status (e.g., married, single), and UK residency.

**Language difficulties.** Participants were asked to report their diagnostic label, that is, whether they had ever received a formal diagnosis. They also indicated the nature of their language difficulties by selecting all relevant difficulties from the following list: receptive language, expressive language, word learning, word finding problems, pragmatic language problems, and articulation problems. These difficulties were carefully chosen to represent the most commonly reported ongoing language challenges in the literature (Bishop et al., 2016, 2017; Botting, 2020; McGregor et al., 2017). They are not intended to be mutually exclusive and sought to cover issues that people with DLD would recognize as difficulties. To aid understanding of these terms, lay descriptions of these problems were also provided (e.g., “using socially appropriate words” for “pragmatic language problems”). The measurement of language difficulties is in addition to the STAND screening questionnaire, which was only administered if participants did not report a DLD diagnosis.

**Co-occurring conditions.** Participants also selected all co-occurring conditions they experienced from the following: depression, anxiety, ADHD, autism, dyspraxia, dyslexia, dyscalculia or “other.” These co-occurrences were chosen to reflect the main emotional and neurodevelopmental conditions in the DSM-5 (APA, 2013) and other literature (e.g., Botting et al., 2016; Cleaton & Kirby, 2018).

#### Yearly Survey

The participants with DLD and our TLD comparison sample completed the items below. The TLD comparison sample also completed age, gender, ethnicity, multilingual status, and country of residence questions from the sign-up questionnaire. Completion of the yearly survey was optional and voluntary for the DLD sample; therefore, there is a reduced amount of DLD participants with this data.

**SES.** Participants reported monthly household income, receipt of financial state benefits, education levels, and employment status.

**Emotional health.** Participants completed several well-known, psychometrically validated scales measuring psychological constructs linked to emotional health. The 9-item depression subscale of the Patient Health Questionnaire (Kroenke et al., 2001) measured **depression,** the generalized anxiety disorder assessment (GAD-7; Spitzer et al., 2006) evaluated **anxiety,** the Rosenberg self-esteem scale (Schmitt & Allik, 2005) tested **Self-esteem,** The new general self-efficacy scale (Chen et al., 2001) measured **self-efficacy,** and **alexithymia** (the ability to identify one's emotions) was assessed using the Toronto alexithymia scale-20 (TAS-20; Bagby et al., 1994). We calculated Cronbach's alpha reliability for each scale for the DLD and TLD group separately. Reliability ranged from acceptable to excellent in both groups for the majority of measures (.78 to .94), with only one in the acceptable range and the remaining in the good to excellent range. The only exception was the externally orientating thinking subscale of the TAS-20, which had questionable reliability in both the DLD (α = .63) and TLD groups (α = .62). We retained the externally orientating subscale due to the exploratory nature of this study. Due to space constraints of the short reporting format, please see a more detailed description of these scales in the E-DLD cohort paper (St Clair et al., 2023).

#### Analysis Plan

Our analysis aims to provide descriptive and comparative insights. In the results, we present descriptive statistics in the form of means and SDs for continuous variables, and N and percentage values for categorical variables. We investigated the comparisons between the total DLD (i.e., “diagnosed” + “possible”) and TLD comparison sample where possible. We also investigated the differences between the “diagnosed” and “possible” DLD groups across the variables. Due to the limited word count in this short report, non-significant differences between the “diagnosed” and “possible" DLD groups are not always reported.

For categorical dependent variables, we use chi-square or Fisher's exact tests, dependent on the level of expected values in each cell. Cramer's V is used as a measure of effect size. For the continuous variables, where there is extreme skew such that the most common value is 0 (e.g., depression and anxiety scores), negative binomial regression is used to evaluate group differences. Where there are no distributional violations, independent t-tests are used. Cohen's *d* is used as the effect size for these comparisons. Where there are assumption violations, robust regression is used to analyze group differences, again with Cohen's *d* as the effect size. For all analyses, we adopt a threshold of *p* < 0.05. Given the exploratory nature of the work, we are not applying adjustments for multiple comparisons.

We had very little missing data for most of our categorical variables, ranging from one participant missing on the parent variable, three participants missing data on the language difficulties and the co-occurring difficulties variables, to two participants missing on the income and education variables. As there was very little missing data and the prediction of categorical demographic data is difficult to extrapolate, we used listwise deletion for the analysis of the categorical variables. The missing data on the emotional health composites ranged from two on the GAD to nine on the TAS-20 Total Score. The total TAS-20 score was the only score we could extrapolate the missing total, as this scale has more than 10 items and seven of the nine participants with missing data answered over 90% of the underlying items. We calculated what the total score would have been for these seven participants had they completed the full scale, using the total score from their reduced responses, dividing this value by the number of their valid responses and then multiplying the resulting figure by the total number of items in the scale. This creates a good estimate of what the total score would have been had the respondents completed all items.

## Results

### Sign-Up Stage Data

Participants in the DLD groups (“Diagnosed DLD” and “Possible DLD”) completed the sign-up questionnaire, and so this section reports only on characteristics of participants with DLD. Table 2 provides an overview of the family circumstances of our participants at the sign-up questionnaire stage. In total, 43.3% of our Total DLD sample were parents, with an average of two children, which did not differ by diagnostic group (*p* = 1.0). When considering the Total DLD sample, 52.8% reported being in a relationship, either living with their partner or separately. A Fisher's exact test showed a significant association with a medium effect size between DLD diagnosis status and relationship status, with those who have diagnosed DLD less likely than those with possible DLD to be living with a partner and more likely to be separated/single or dating/living apart (*p* = .013, V = .32).

#### Language Difficulties

The most reported language difficulty among the combined DLD group at the sign-up stage (see Table 3) was word finding difficulties, followed by difficulties with expressive language, receptive language, and then word learning. Less common were pragmatic language and articulation difficulties. Of note is that pragmatic language difficulties were significantly more common in those with diagnosed DLD than those with possible DLD (χ^2^ (1, 88) = 7.54, *p* = .006, V = .29). No other domain had significant differences between the Diagnosed and Possible DLD (*p*s between .304 and 1.0).

**Table 3.** Self-Reported Areas of Language Difficulty in Adults with DLD.

	Diagnosed DLD (N = 28)	Possible DLD (N = 60)	Total DLD (N = 88)
Word finding	25 (89.3%)	54 (90.0%)	79 (89.8%)
Expressive language	24 (85.7%)	55 (91.7%)	79 (89.8%)
Receptive language	23 (82.1%)	44 (73.3%)	67 (76.1%)
Word learning	19 (67.9%)	38 (63.3%)	57 (64.8%)
Pragmatic language	20 (71.4%)	24 (40.0%)	44 (50.0%)
Articulation	11 (39.3%)	17 (28.3%)	28 (31.8%)

*Note.* DLD = developmental language disorder.

#### Co-Occurring Difficulties

Anxiety was the most prevalent co-occurring condition for adults with DLD, followed by depression, ADHD, and dyslexia. Individuals with diagnosed DLD were more likely to report a diagnosis of dyslexia with a small effect size (χ^2^ (1, 88) = 5.38, *p* = .02, V = .25). There were no other differences between the Diagnosed DLD and the Possible DLD groups (*p*s between .360 and .931). Just a handful of dyscalculia and dyspraxia diagnoses were represented (see Table 4). A small number (n = 14) of our sample also reported that they had autism. Although this would exclude them from DLD diagnosis when strictly applying current diagnostic criteria (Bishop et al., 2016), we recognize that the pathways and diagnostic boundaries of communication difficulties are complex (Thomas et al., 2019), and thus we did not exclude these individuals from the present sample because their DLD was reported as the primary difficulty, and because participants were mostly diagnosed before current DLD criteria were established.

**Table 4.** Self-Reported Additional Diagnoses of Adults with DLD.

	Diagnosed DLD (N = 28)	Possible DLD (N = 60)	Total DLD (N = 88)
Anxiety	14 (50.0%)	32 (53.3%)	46 (52.3%)
Depression	10 (35.7%)	22 (36.7%)	32 (36.4%)
ADHD	8 (28.6%)	18 (30.0%)	26 (29.6%)
Dyslexia	11 (39.3%)	10 (16.7%)	21 (23.9%)
Autism	6 (21.4%)	8 (13.3%)	14 (15.9%)
Dyscalculia	3 (10.7%)	3 (5.0%)	6 (6.8%)
Dyspraxia	2 (7.1%)	3 (5.0%)	5 (5.7%)

*Note.* DLD = developmental language disorder.

### Yearly Survey Data

Overall, 30 adult DLD participants also completed the voluntary yearly survey. The same TLD comparison group as described above also completed these items.

#### Education and Employment

There were no significant differences in educational qualifications (χ^2^ (2, 118) = 0.98, *p* = .61, V = .09), employment outcomes (Fisher's exact *p* = .31, V = .17), or being in receipt of state financial benefits (Fisher's exact *p* = .13, V = −.15) between the DLD and the TLD samples.

There was a significant difference with a medium effect size in monthly net income between the DLD and the TLD sample (Fisher's exact *p* = .007, V = .36; see Table 5), with the DLD sample over-represented on the lower end of the income scale, while the TLD group was more likely to have higher income. Note, however, that the DLD group is represented in all income bands, including the highest, and that relative salary will differ across different countries.

**Table 5.** Education and Employment Characteristics of DLD Samples and TLD Comparison Sample.

	Diagnosed DLD (N = 12)	Possible DLD (N = 18)	Total DLD (N = 30)	TLD Comparison (N = 91)
Monthly household net income^a^				
Under £1000	3 (27.3%)	6 (35.3%)	9 (32.1%)	7 (7.7%)
£1001-£2000	3 (27.3%)	6 (35.3%)	9 (32.1%)	20 (22.0%)
£2001-£3000	3 (27.3%)	1 (5.9%)	4 (14.3%)	21 (23.1%)
£3001-£4000	1 (9.1%)	3 (17.7%)	4 (14.3%)	20 (22.0%)
Over £4001	1 (9.1%)	1 (5.9%)	2 (7.1%)	23 (25.3%)
Highest educational qualification^b^				
<= Level 3 qualification	5 (41.7%)	3 (17.7%)	8 (27.6%)	19 (20.8%)
Level 5 or 6 qualification	4 (33.3%)	8 (47.1%)	12 (41.4%)	48 (52.8%)
Level 7 or above qualification	1 (8.3%)	6 (35.3%)	7 (24.1%)	24 (26.4%)
Not reported	2 (16.7%)	–	2 (6.9%)	–
Employment^c^				
Full time job	4 (33.3%)	9 (50.0%)	13 (43.3%)	45 (50.6%)
Part-time job	3 (25.0%)	5 (27.8%)	8 (26.7%)	19 (21.4%)
Unemployed	4 (33.3%)	3 (16.7%)	7 (23.3%)	24 (27.0%)
Unemployed, in education	1 (8.3%)	1 (5.6%)	2 (6.7%)	1 (1.1%)
Receiving state benefits	4 (33.3%)	3 (16.7%)	7 (23.3%)	10 (11.0%)

*Note.* No comparisons between the diagnosed and possible DLD groups were significant. DLD = developmental language disorder; TLD = typical language development.

a There were two missing values in the DLD group, one in the Diagnosed DLD and on in the Possible DLD subgroups.

b There was one missing value in the possible DLD group.

c There were two missing values in the TLD comparison group.

#### Emotional Health

Individuals with DLD had higher depression and anxiety symptoms and were more likely to be above the clinical cutoff for these measurements (see Table 6). The DLD sample also had lower self-esteem and self-efficacy scores, and increased rates of alexithymia. They had a higher total score than the TLD group, were more likely to be above the cutoff to be considered as having alexithymia and had higher scores on all subscales, but the difference in the externally orientated thinking subscale was just off significance. Where it was possible, effect sizes were calculated and ranged from medium to large, with the exception of the effect size for the total alexithymia score being very large, and the effect size for the alexithymia subscale of externally orientated thinking was small, which is consistent with this comparison being non-significant.

**Table 6.** Emotional Health Measures for the DLD Sample and the TLD Comparison Group.

	Diagnosed DLD (N = 12)	Possible DLD (N = 18)	Total DLD (N = 30)	TLD Comparison (N = 91)	Comparison (Total DLD Vs TLD)
Depressive symptoms^a^ (M(SD))	13.73 (5.93)	12.56 (7.39)	13.00 (6.79)	5.00 (5.24)	B = −.95, 95%CI[-1.33,−.58], *p* < .001
N (%) above cutoff ^b^	8 (72.7%)	11 (61.1%)	19 (65.3%)	13 (15.3%)	χ^2^ (1, 114) = 27.01, *p* < .001, V = −.50
Anxiety symptoms^a^ (M(SD))	12.42 (5.50)	9.89 (6.41)	10.90 (6.09)	4.24 (4.37)	B = −.95, 95%CI[-1.37,−.52], *p* < .001
N (%) above cutoff ^b^	8 (66.7%)	9 (50.0%)	17 (56.7%)	11 (12.4%)	χ^2^ (1, 119) = 24.48, *p* < .001, V = −.45
Self-esteem (M (SD))^c^	23.92 (4.50)	23.78 (5.87)	23.83 (5.36)	29.90 (6.18)	*t* (113) = -4.72; *p* < .001, *d* = -1.01
Self-efficacy^d^	25.82 (6.38)	23.29 (5.99)	24.29 (6.16)	30.22 (5.28)	B = 5.93, 95%CI[3.39, 8.49]. *p* < .001, *d* = -1.08
Alexithymia Total score (M(SD))^d^	65.25 (9.31)	66.77 (10.08)	66.12 (9.61)	52.27 (9.14)	B = -13.85, 95%CI[-17.89,-9.81], *p* < .001, *d* = 1.50
N (%) above cutoff^b^	8 (66.7%)	12 (75.0%)	20 (71.4%)	17 (18.7%)	χ^2^ (1, 119) = 27.81, *p* < .001, V = −.48
Alexithymia Externally orientated thinking subscale^c^	26.5 (3.75)	27.60 (3.48)	27.11 (3.58)	25.76 (3.11)	*t* (113) = 1.90; *p* *=* .06, *d* = .42
Alexithymia difficulty identifying emotions subscale^a^	22.33 (5.63)	22.69 (6.45)	22.54 (6.00)	13.17 (5.78)	B = −.54, 95%CI[−.69,−.38], *p* < .001
Alexithymia difficulty describing feelings subscale^d^	16.42 (2.64)	16.73 (3.45)	16.59 (3.07)	13.42 (3.34)	B = -3.17, 95%CI[-4.52,-1.82], *p* < .001, *d* = .97

*Note.* DLD = developmental language disorder; TLD = typical language development. No comparisons between the diagnosed and possible DLD groups were significant. There was a small amount of missing values in these measures, detailed as follows: depressive symptoms—one participant from the diagnosed DLD group and six from the TLD group; anxiety symptoms—two participants from the TLD group; self-esteem—one participant from the diagnosed DLD group and five from the TLD group; self-efficacy—one from the diagnosed DLD group, one from the possible DLD group, four from the TLD group; alexithymia total score—two from the possible DLD group; alexithymia externally orientated thinking subscale—three from the possible DLD group, three from the TLD group; alexithymia difficulty identifying emotions subscale—two from the possible DLD group, three from the TLD group; alexithymia difficulty describing feelings subscale—three from the possible DLD group, one from the TLD group.

a Negative Binomial regression was used, as there was extreme skew in the data.

b Chi-square tests were used on this categorical data.

c Independent samples t-tests were used as no assumptions were violated.

d Robust regression was used as assumptions were violated.

## Discussion

This short report presents important information about adults with DLD provided by the adults themselves, to inform awareness and support for this community. In this discussion, we reflect on some of the key findings with reference to the current literature, to contribute to the small but emerging evidence base to inform the work of clinicians and researchers.

### Expressive and Receptive Language Difficulties

The most prevalent self-reported areas of language difficulty in the combined “diagnosed” and “possible” DLD samples (“Total DLD” in the tables above) were word finding and expressive difficulties. This could reflect the more “visible” nature of expressive language impairments and echoes referral patterns observed in childhood where parents have been found to detect expressive difficulties more readily than receptive difficulties (Hayiou-Thomas et al., 2014).

A substantial proportion of the diagnosed DLD and possible DLD groups reported receptive language difficulties (respectively, 82.1% [23/28] and 73.3% [44/60]). The difference between “diagnosed DLD” respondents and the “possible DLD” group was not significant for either receptive or expressive difficulties. Nevertheless, from a clinical perspective, it is interesting to consider the possibility that additional diagnostic reports may help to build insight into those aspects of language disorders that are less immediately evident. Furthermore, it may be the case that receptive language difficulties are associated with higher levels of functional impairment that highlight a clear need for language assessment and support. While further research is needed, these insights could guide future development of self-identification tools, perhaps through work to understand the lived experiences of those with receptive difficulties. This tool could then be shared with others concerned about their communication, but who may not understand that the root of day-to-day challenges they face could lie in comprehension or processing challenges.

### Pragmatic Language Difficulties

Self-reported pragmatic language difficulties were more prevalent in the formally diagnosed sample. This may indicate that language difficulties are more readily apparent—thus prompting individuals to seek a diagnosis—when accompanied by social pragmatic difficulties, due to an additive effect and potential functional impact (Mueller & Tomblin, 2012). Another explanation may lie in the close relationship between structural language competence and pragmatic language competence (Gibson & St Clair, 2020): Those clinically diagnosed with DLD could plausibly be those with more severe structural language impairments. However, the latter interpretation is complicated by potential SES effects—access to diagnosis could be linked to affluence rather than severity of need (Bishop & McDonald, 2009). Unlike recent systematic reviews (e.g., Ziegenfusz et al., 2022), the present study did not find differences in education, take-home pay, or employment between our “diagnosed” and “possible” DLD groups. It is important to note that our sample is self-selecting and therefore may represent a higher achieving subgroup in this respect. Furthermore, our analyses are insufficiently powered to properly examine predictors of differences, and further research is needed to investigate these fully. Overall, these results suggest the need for comprehensive language assessment for adults concerned about their communication, rather than a focus on single areas like word finding or word learning. However, this is complicated by the fact that many language assessments are not normed for adults.

### Emotional Wellbeing

The findings on emotional wellbeing are consistent with the wider body of literature on DLD and mental health (e.g., Botting & Conti-Ramsden, 2008; Botting et al., 2016; Conti-Ramsden & Botting, 2008; Nudel et al., 2023). The adults in our DLD samples had higher scores for measures of depression and anxiety and were more likely to have scores above clinical thresholds for diagnosis. Furthermore, these adults also had poorer scores on the alexithymia, self-esteem and self-efficacy scales, showing difficulties that have been linked to poor emotional health (St Clair et al., 2023). These difficulties may arise because of the impact of DLD on daily life; may link to difficulties with learning emotional vocabulary, talking through feelings and problem solving; or may be part of a neurological profile that includes emotional as well as communicative differences. These findings also hold importance for psychological wellbeing practitioners, aligning with recent research highlighting the need to cater for individuals with language and communication difficulties, including individuals with DLD, within psychological services (Hancock et al., 2023).

### Participant Profiles

Another finding of note is that most of the DLD adult respondents were women. This is inconsistent with childhood-based studies, where boys are more likely than girls to be diagnosed with language difficulties (Chilosi et al., 2023; Norbury et al., 2016). However, this finding is consistent with the recent uptick in women being identified as being neurodivergent in adulthood, attributed to a lack of research and clinical attention to neurodevelopmental profiles in girls (Harrop et al., 2024). It is also the case that women are more likely to participate in survey-based research (Nuzzo & Deaner, 2023). Participants with DLD were just as likely to be employed, on benefits or in education as our TLD participants. This is consistent with our expectations based on previous research (Conti-Ramsden et al., 2018; Dubois et al., 2020), and it is encouraging to see that some individuals with DLD are experiencing similar outcomes to peers. Nevertheless, a limitation of this study is the relatively crude categories of education used, with Further Education and Higher Education grouped together, and the self-selected sample, which may attract more affluent or successful adults with DLD. However, aligning with some previous findings, we see that those with DLD are more likely to earn less than TLD peers (Beitchman et al., 2014; although note that findings are inconsistent: see Dubois et al., 2020 for a review). This may be because they are in roles that require fewer qualifications or perhaps because they face systemic barriers to workplace progression opportunities or even that they are less likely or able to advocate and negotiate a pay rise. It is also encouraging to see representation of those with DLD across the income banding. However, income was not standardized across different countries in this study, and a more detailed analysis is warranted in future large-scale multinational studies.

### Limitations and Future Directions

The work we have reported here has some limitations to note. Perhaps most significantly, the TLD sample was recruited via an online platform with recruitment occurring during the day. This may have biased our comparison group toward those less likely to be at work, meaning we may have overestimated the employment challenges of those in the TLD group. Relatedly, we also note that the DLD samples here are relatively small and may not be representative of the wider global population in terms of ethnicity and languages spoken. Additionally, as the DLD sample had signed up for the E-DLD project, we may over-represent more affluent adults with DLD, who have access to devices and the time to sign-up to this project. Furthermore, our DLD groups are based on self-reported data and a screening questionnaire which is in the process of being validated. Although this measure shows highly promising initial results (St Clair et al., in preparation), more work will be needed to understand how to reach more diverse samples to participate in adult DLD research and to validate self-reports against gold-standard speech and language therapy assessments. As such the current short report should be considered a useful but not conclusive account of work in progress. It is intended to raise awareness of the long-term impact of DLD to inform accommodations and support for this community. We hope the profiles reported in our paper will support speech and language therapists in joining DLD advocates in the call for better recognition and support.

### Conclusion and Implications

Overall, the E-DLD cohort shows that there is a need for research and clinical attention to DLD in adults. Despite the condition not being as widely known as other neurodevelopmental needs (McGregor, 2020), the project was successful in recruiting a sample of those who had a clinical diagnosis as well as a sample of those who consider themselves to have DLD. We identified that expressive language and word finding difficulties are reported by over 90% of those adults who think they may have DLD and conclude that this observation could feed into future awareness raising efforts. Mental health difficulties were a defining feature of the DLD group, indicating that more research needs to go into looking at the needs of adults with DLD in accessing mental health support. We found that those with DLD were disproportionately represented in the low-income category and recommend that services respond by improving access to affordable care. We hope that the profiles shared here will influence the setup of services and future research studies.

The overarching change we hope to see is better recognition of DLD in adults, leading to effective workplace/manager training, better understanding and implementation of reasonable adjustments for adults with DLD in accordance with equalities legislation, and widespread access to diagnostic clinics and support professionals.

## Supplemental Material

sj-docx-1-dli-10.1177_23969415261461413 - Supplemental material for Who are Adults with Developmental Language Disorder? Profiles from the Engage with Developmental Language Disorder Project Supplemental material, sj-docx-1-dli-10.1177_23969415261461413 for Who are Adults with Developmental Language Disorder? Profiles from the Engage with Developmental Language Disorder Project by Jenny L Gibson, Nicola Botting, Ellie Barker, Emily M Jackson, Suze Leitão and Michelle C St Clair in Autism & Developmental Language Impairments

## Notes

1. For ease of reading, we use the term DLD where authors may have used other terminology such as Language Impairment (LI), Specific Language Impairment (SLI) or Language Disorder (LD) in their texts.
