# Supplementary material for: Who are Adults with Developmental Language Disorder? Profiles from the Engage with Developmental Language Disorder Project
Source: Autism Dev Lang Impair. 2026 Aug 10;11:23969415261461413. doi: 10.1177/23969415261461413 (PMC13458118; doi:10.1177/23969415261461413)
Supplement: Supplementary file 1 [file sj-docx-1-dli-10.1177_23969415261461413.docx]

Table 1. Demographic information for those with Diagnosed DLD, Possible DLD, the combined DLD group and TLD comparison group who completed yearly surveys.

|  | Diagnosed DLD (N = 12) | Possible DLD (N = 18) | Total DLD (N = 30) | TLD Comparison (N = 91) |
| --- | --- | --- | --- | --- |
| Gender* (N (% female)) | 11 (91.7%) | 11 (61.1%) | 22 (73.3%) | 60 (65.9%) |
| Age (M, SD) | 42.08 (14.27) | 39.39 (14.27) | 40.47 (13.07) | 42.83 (13.6) |
| Ethnicity (N, %) |  |  |  |  |
| White | 12 (100.0%) | 13 (72.2%) | 25 (83.3%) | 76 (83.5%) |
| Asian | -- | 1 (5.6%) | 1 (3.3%) | 7 (7.7%) |
| Black | -- | 1 (5.6%) | 1 (3.3%) | 3 (3.3%) |
| Mixed | -- | -- | -- | 3 (3.3%) |
| Hispanic | -- | -- | -- | 1 (1.1%) |
| Other, not reported | -- | 3 (16.7%) | 3 (10.0%) | 1 (1.1%) |
| Bilingual Status (N, %) |  |  |  |  |
| Monolingual | 11 (91.7%) | 13 (72.2%) | 24 (80.0%) | 80 (87.9%) |
| Bilingual | 1 (8.3%) | 4 (22.2%) | 5 (16.7%) | 11 (12.1%) |
| Multilingual | -- | 1 (5.6%) | 1 (3.3%) | -- |
| UK Resident^1^ (N, %) | 9 (75.0%) | 13 (76.5%) | 22 (75.9%) | 91 (100%) |

^1^One participant in the Possible DLD group have missing data on the UK residency variable.
